# Interference with the Autophagic Process as a Viral Strategy to Escape from the Immune Control: Lesson from Gamma Herpesviruses

**DOI:** 10.1155/2015/546063

**Published:** 2015-05-18

**Authors:** Roberta Santarelli, Marisa Granato, Alberto Faggioni, Mara Cirone

**Affiliations:** Department of Experimental Medicine, “Sapienza” University of Rome, Viale Regina Elena 324, 00161 Rome, Italy

## Abstract

We summarized the most recent findings on the role of autophagy in antiviral immune response. We described how viruses have developed strategies to subvert the autophagic process. A particular attention has been given to Epstein-Barr and Kaposi's sarcoma associated Herpesvirus, viruses studied for many years in our laboratory. These two viruses belong to *γ*-Herpesvirus subfamily and are associated with several human cancers. Besides the effects on the immune response, we have described how autophagy subversion by viruses may also concur to the enhancement of their replication and to viral tumorigenesis.

## 1. Autophagy and the Immune Response

According to the paradigm of antigen processing by the antigen presenting cells (APC), peptides arising from intracellular proteins are presented via class I MHC molecule. This occurs after they are degraded via proteasome and transported by transporters associated with antigen processing (TAPs). Differently, peptides that originate from extracellular antigens are delivered to the late endosomal/lysosomal compartment, where they are degraded by the lysosomal proteases and then presented in association with class II MHC. In the lysosomes, the invariant chain, which blocks class II MHC, is also degraded, rendering the MHC molecules available for peptide loading. Besides the differences in the sites of origin and processing, peptides presented via class I or II MHC activate different populations of T lymphocytes, which are CD8^+^ or CD4^+^, respectively [1]. However, exceptions to this model have been described and, for example, dendritic cells (DCs), the most powerful APC [2], are able to present extracellular antigens also via class I MHC. This event, known as cross-presentation, allows DCs to activate both CD4^+^ and CD8^+^ T cells, in response to an extracellular antigen [3]. On the other hand, antigens of intracellular origin can be presented also in class II MHC, after being delivered to the lysosomes, through double membrane vesicles, called autophagosomes [4]. Autophagosomes are formed during the induction of autophagy, a self-eating mechanism through which cells recycle their own constituents and survive in stressful conditions. This represents one of the mechanisms through which autophagy may promote the immune response. The autophagic process is regulated by AuTophaGy-related (ATG) genes [5]. Some of them, such as Atg1, Atg11, and Atg13, regulate autophagosome formation; others (e.g., Atg2, Atg9, and Atg18) are required for membrane flow to the expanding phagophore. The vesicle nucleation is instead dependent on the class-III phosphatidylinositol 3 kinase (PtdIns3K) complex formed by Vps34, Vps15, Vps30/Atg6, and Atg14. Atg6, also named Beclin 1, represents a protein with a pivotal role in the autophagy induction [6] and the microtubule-associated protein light chain 3 (LC3), or Atg8, is a marker of the autophagic vacuoles [7]. LC3 is expressed as full-length cytosolic protein and upon autophagy induction is cleaved by Atg4 to form LC3I, which is then conjugated to phosphatidylethanolamine (PE), generating LC3II. This molecule, whose formation indicates autophagy induction, is associated with the internal and external membrane of autophagosome and, by interacting with adaptor molecules, such as p62/SQSTM1, promotes uptake and degradation of both cargo and adaptors into the lysosomes [8]. Indeed, autophagosomes fuse with lysosomes transporting intracellular proteins in the last autophagic steps. Among them, also viral antigens can end up in the lysosomes, in virally infected cells. Here peptides derived from their degradation may be complexed and presented in association with class II MHC molecules. Another important role of autophagosomes is to transport entire viral particles to the endosomal/lysosomal compartment, to be degraded and eventually eliminated, a process known as xenophagy [9]. After entering the cells, pathogens can be directly delivered to the lysosomes through phagosomes. Alternatively, phagosomes as well as free viral particles can be engulfed by the double membrane vesicles of autophagosomes, before reaching the lysosomes. The latter process is under the control of the ATG genes, even if it represents a particular form of autophagy aimed at the antimicrobial defense [10]. In addition, autophagy facilitates the pathogen engagement of intracellular TLRs and the consequent release of cytokines, such as type I IFN [11]. The binding of TLRs as well as other molecules involved in the immune response, for example, CD46, by ligands or pathogens that use them as receptors, also triggers autophagy. This has been reported for Epstein-Barr virus (EBV) that binds TLRs [12] and for measles virus that uses CD46 as receptor [13]. Autophagy has been also shown to contribute to measles virus infectivity [14]. Intriguingly, since CD46 represents the cellular receptor also for human Herpes-virus 6 (HHV6) [15], it would be interesting to investigate the impact on autophagy of CD46 engagement by HHV6. As if the role of autophagy in the immune response was not enough important, it has been recently reported that autophagy is induced by GM-CSF in monocytes and that it stimulates monocyte differentiation into functional macrophages and DCs. Besides that, autophagy also plays an essential role in preventing apoptosis of these cells [16].

## 2. Autophagy Manipulation by Viruses

**Figure 1:**
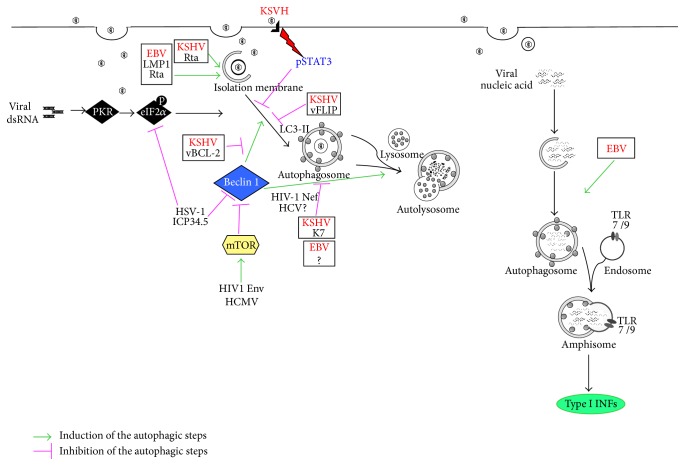
Examples of autophagy manipulation by human viruses.

Among the strategies that allow viruses to escape from the immune control, the impairment of monocyte differentiation into functional DCs represents a common one [17–19]. Based on the recent finding, suggesting that autophagy is involved in monocyte differentiation in macrophages and DCs, the interference with the autophagic process could represent one of the underlying mechanisms responsible for such impairment. This strategy has been recently reported to be exploited by Human Hepatitis C virus (HCV), whose infection of human monocytes results in a reduction of lysosomal cathepsins, in an autophagic block at the late steps, and, as a consequence, in an impairment of DC differentiation [20]. HCV-mediated reduction of lysosomal acidification, as result of cathepsin release, has been reported also in other cell types [21]. However, autophagy can contribute to HCV replication since the autophagosomal membranes can be used for viral production [22]. Given the importance of autophagy in the immune response, it is not surprising that viruses have evolved strategies to interfere with it, in order to avoid their elimination into the lysosomes, impair the production of antiviral cytokines, reduce the presentation of their antigens, and, as described for HCV, alter DC differentiation. This is a must for viruses to persist in the infected host, sometimes with pathological consequences. All the steps of the autophagic process, from the autophagosome formation to the lysosomal degradation of their content [23], can be manipulated by viruses [24, 25] (Figure 1). It has been described that it depends on the virus types, on the phase of their life cycle, and on the host cell that they infect. For example, Human Immunodeficiency Virus [23], a single-stranded RNA lentivirus belonging to the* Retroviridae* family, is able to induce the initial phases of autophagy by Env, an envelope fusogenic protein. On the other hand, it blocks the late autophagic phases to enhance viral production by expressing Nef, an accessory protein that interferes with the autophagosomal maturation [24, 25]. During the replicative phase of their life cycle, also herpesviruses such as EBV and Kaposi's Sarcoma Human Herpesvirus (KSHV) can promote autophagy and exploit the autophagic machinery to enhance their replication [26–28]. To do so, they promote the first autophagic steps and block the last ones. This strategy allows them to avoid being delivered into the destructive environment of the lysosomes and to usurp the autophagic machinery for viral transportation through the cell cytoplasm [22, 26, 29]. Pathways involved in the autophagy induction, such as PKR/EIF2 alpha and m-Tor, can be also targeted by viruses belonging to the Herpesvirus family, for example, Herpes Simplex virus-1 (HSV-1) [30–32] and cytomegalovirus (HCMV) [33]. Also Beclin 1, a key molecule involved in several steps of the autophagic process [6], can be bound and altered in its function by viral proteins such as vBcl2 of KSHV or ICP 34.5 of HSV-1 [34–36]. All these strategies allow viruses to block autophagy induction during infection of the host cells. Differently, another Herpesvirus, Varicella-Zoster virus (VZV), has been recently reported to successfully infect target cells without blocking the autophagic process. Indeed ICP 34.5 or US11, the two proteins responsible for the block of autophagy by HSV1, are not present in VZV although both viruses belong to the same subfamily [37].

## 3. Herpesviruses

Herpesviruses are large, double strand DNA viruses having a common particle structure [38]. To date eight human Herpesviruses have been identified and classified into three subfamilies (alpha, beta, and gamma) based on their growth characteristics and tissue tropism [39]. The *α*-subfamily includes the neurotropic viruses Herpes Simplex viruses (HSV) 1 and 2 and Varicella-Zoster virus (VZV). Human cytomegalovirus (HCMV) and the human Herpesviruses 6 and 7 are members of the *β*-subfamily, while EBV and KSHV are members of the *γ*-subfamily [40]. A common feature shared by all human Herpesviruses is the viral persistence into host and the possibility to undergo two alternative life cycle programs, namely, latency and lytic replication. During latency the viral genome is retained as a circular episome in the nucleus and no viral progeny is produced. Furthermore, in the course of latent infection a limited set of genes is expressed in order to reduce immune recognition. In contrast to latency, the Herpesvirus lytic program is characterized by a regulated cascade of viral gene expression accompanied by viral production and killing of infected cells [23]. Although most Herpesviruses have been reported to interfere with the autophagic process, the next part of this review will focus on the interplay between autophagy and *γ*-Herpesviruses, given that our laboratory has for long time worked on the virus-host interaction of these viruses associated with several human cancers.

## 4. Epstein-Barr Virus (EBV)

As for other Herpesviruses, EBV infection can be latent or lytic [41, 42]. During latency the EBV genome is retained as a circular episome in the cell nuclei and upon appropriate stimuli the virus switches into the lytic replication program. It is known that EBV infects primarily human B lymphocytes and epithelial cells; however, its infection can also occur in other cells with a central role in the immune response [43], such as monocytes and DCs [44, 45]. The* in vitro* infection of monocytes induces apoptosis and results in an impairment of DC development [45, 46], although the underlying mechanisms have not been investigated in these studies. Therefore, the autophagic pathway could be investigated in these cells since, as previously described, a block of autophagy can switch cell differentiation into cell death in monocytes DCs [16]. These are the most powerful cells in the priming of the CD8^+^ T cell response, playing a pivotal role in control of the EBV infection [47]. Besides cytotoxic T cells, DCs are able to activate a CD4^+^ T cell-mediated immune response against the EBV antigens, such as EBNA1, through the mechanism of cross-presentation [48]. Autophagy has been shown to be essential for class II MHC presentation of EBNA1 protein. The block of autophagy resulted in an accumulation of this protein in the intracellular autophagosomes and, more importantly, in a reduction of EBNA1 recognition by EBNA1 specific CD4^+^ T cells [49]. The involvement of autophagy in EBNA 1 presentation was later confirmed by a more recent study [50]. Regarding the EBV interaction with plasmacytoid subpopulation of DCs (pDCs), the main type I IFN producing cells [51], it has been reported that autophagy is essential for IFN release in response to the EBV infection. Autophagy is stimulated by the virus and facilitates its interaction with TLR7 and TLR9 PPRs, both located in the endosomal/lysosomal compartment and essential for EBV recognition by these cells. However, although EBV stimulates autophagy and IFN release, its infection results in an impairment of pDC maturation [12]. Since it has been reported that EBV downregulates TLR 9 in B cells [52] and that this effect is mediated by two EBV proteins, namely, latent membrane protein 1 (LMP1) [53] and BGLF5 [54], it would be interesting to investigate TLR9 expression levels in the pDCs EBV-infected versus uninfected control cells.

EBV is associated with several different human cancers of B and epithelial cell origin, such as posttransplant lymphoproliferative disorder (PTLD), Hodgkin and non-Hodgkin lymphomas, nasopharyngeal carcinoma (NPC), and some forms of gastric carcinoma [55]. Besides its strong association with some human cancers, EBV infects and establishes a life-long asymptomatic infection in 95% of adult healthy population [56], reducing to the minimum or not expressing any protein, in order to escape from the immune recognition. Among the viral antigens, only the EBV latent nuclear antigen 1 (EBNA1) expression is required for the maintenance of the viral episome in the EBV infected human B cells [57] and this is the only protein always expressed in EBV-associated malignancies. This protein has been demonstrated to activate both CD4^+^ T cells [44], being presented via class II MHC [49] and CD8^+^ T cells [58], although for long time it has been considered invisible to the immune system [48]. As previously described, autophagy is essential for EBNA1 antigen presentation via class II MHC while, interestingly, it does not seem to play a role in the class II MHC presentation of two other EBV latent nuclear proteins, EBNA2 and EBNA 3C, both expressed only in pathological conditions [59]. The most oncogenic EBV latent protein, LMP1, has been reported to regulate its own expression by inducing or inhibiting autophagy. When LMP1 is highly expressed in B cells, autophagy is stimulated and promotes degradation [60]. Given that LMP1 plays an important role in EBV-induced oncogenesis, the stimulation of autophagy could be used as a strategy to reduce the expression of this protein and consequently to affect the EBV-driven tumorigenesis.

## 5. Kaposi's Sarcoma-Associated Herpesvirus (KSHV)

KSHV or Human Herpesvirus-8 (HHV-8) is the last human *γ*-Herpesvirus identified to date [61]. It shows two alternative life cycle programs, latent and lytic [62–64]. Generally, latency is the KSHV default program within 48–72 h postinfection depending on target cells.* In vivo*, KSHV mainly infects endothelial and B cells and establishes a lifelong latency in B lymphocytes of infected individuals, escaping from the host immune response [65, 66]. Several KSHV latent and lytic proteins are involved in immune evasion, in preventing apoptosis and blocking autophagy as well as in transformation [67]. KSHV is the etiologic agent of Kaposi's sarcoma (KS) [61], a multifocal angioproliferative disorder arising from KSHV-infected endothelial cell [68], and of lymphoproliferative disorders, such as primary effusion lymphomas (PELs), a non-Hodgkin B cell lymphoma localized in body cavities [66], and the plasma cell variant of multicentric Castleman disease (MCD) [69]. It is important to underline that, although KSHV is detectable in all KS lesions, regardless of disease stage or clinical variant, the virus is necessary but not sufficient for the development of this tumor [67, 68]. Typical of KS are the spindle cells, derived from the endothelial cells. The majority of spindle cells are latently infected by KSHV and only a small percentage of these cells express viral lytic antigens [68, 70]. However, it is currently believed that lytic replication is necessary to support KS lesion formation and maintenance [68, 71–74]. In addition, viral replication allows the secretion of proinflammatory and/or proangiogenic factors that create the inflammatory microenvironment essential in KS pathogenesis [68, 75]. Monocytes are among the inflammatory cells found in KS lesions and a study, based on immunohistochemical staining and in situ hybridization, reported that these cells are KSHV infected [76]. The virus has also been detected in peripheral T-cells and macrophages [77, 78].

As other Herpesviruses, KSHV has to cope with innate and adaptive immunity in order to establish a persistent infection in immunocompetent host. Latency is one of the strategies used by KSHV since viral gene expression is reduced. Conversely, during its lytic cycle, a high number of immunogenic proteins are produced [71, 79]. The relevance of the equilibrium between KSHV lytic cycle and host immunity is indicated by the increased viral replication and the development of KSHV-related malignancies in immunosuppressed patients [80–82]. About 25% of KSHV proteins are involved in KSHV immune evasion mechanisms [79] and, although most of these belong to lytic cycle, both latent and lytic proteins are able to hijack innate and adaptive immune response [67]. They can indeed inhibit complement-mediated lysis of infected cells and the IFN I signaling, deregulate the inflammatory cytokine/chemokine networks, and interfere with antigen presentation [67, 79, 83–85]. pDCs, monocyte-derived dendritic cells, and monocytes are among the cell types infected by KSHV [19, 86, 87]. As consequence of the infection, a reduction of costimulatory molecules as well as a deregulation of the cytokine release and an impairment of allostimulatory capacity can occur [19, 76, 87–90].* In vivo*, DC functional impairment has been reported in patients with classical KS [91] and a reduction of pDCs was observed in AIDS-KS in comparison to KS negative HIV-1 infected individuals [92]. Moreover, the number of Langerhans cells is also decreased in KS lesions compared to normal skin [93]. Furthermore, it has been reported that the signal transducer and activator of transcription 3 (STAT3) activation in noninfected monocytes/macrophages leads to a block of autophagy and consequent dysfunction, due to the cytokines released during HIV infection [94]. Interestingly, STAT3 inhibitors are potent autophagy inducers [95]. In this context we showed that KHSV is able to activate STAT3 pathway in DCs by binding to its receptor on these cells, namely, the type II C-type lectin, dendritic cell-specific ICAM-3 grabbing nonintegrin (DC-SIGN; CD209) [87, 96]. Besides, we showed that STAT3 activation led to a block of the autophagic flux, as demonstrated by the reduced expression of LC3II and the increased level of p62 [96]. In addition, looking for a possible mechanism responsible for the block of autophagy mediated by STAT3 activation in DCs exposed to KSHV, we found an upregulation of Mcl-1 [96, 97]. This is one of the proteins able to bind and sequester Beclin 1, hampering its essential role in autophagosome formation. Remarkably, STAT3 inhibition by AG490 was able to prevent the effects on p62, LC3II, and Mcl-1 [96]. In agreement, a previous paper has shown that the inhibition of STAT3 by Sorafenib resulted in a downregulation of Mcl-1, disruption of Beclin 1-Mcl-1 complex, and reversion of the autophagic block in hepatocarcinoma cells [98]. Of note, the autophagic block occurred also in the presence of UV-inactivated KSHV [96], indicating that neither vBCL2 nor vFLIP expression was required [34, 96, 99, 100]. Concomitantly to the autophagic block induced by KSHV-mediated STAT3 activation, we also observed a reduction of IL12p70 release in response to LPS stimulation and a higher production of IL-10, IL-6, and IL-23 [96]. This cytokine pattern skews the Th1/Th2 profile towards Th2 [88] and/or Th17 [101], promoting immunosuppression and inflammation. Therefore, STAT3 activation could be one of the molecular mechanisms underlying KSHV-mediated immunosuppression in DCs. Indeed, STAT3 activation correlates with an immunosuppressive phenotype and DC dysfunction in the KS microenvironment [102] and in the peripheral blood of tumor bearing patients [103, 104]. Several papers have investigated the interplay between KSHV and the autophagic pathway [79]. During the latent phase of its life-cycle, KSHV expresses the FADD-like interleukin-1 beta-converting enzyme inhibitory protein (v-FLIP), a truncated homolog of the cellular FLIP, which besides having an antiapoptotic activity [105, 106] has been recently shown to block the autophagic flux. It competes with LC3 for binding to ATG3, thereby preventing LC3 binding and processing during autophagosome biogenesis [100]. Furthermore, the inhibition of vFLIP binding to ATG3 reduced the size of KSHV positive tumor in mice [100]. It has also been demonstrated that v-FLIP suppression of autophagy counteracts v-cyclin-induced cellular senescence [107, 108]. v-cyclin is a KSHV latent protein that deregulates cell cycle, causes aberrant host DNA replication, and triggers the DNA damage responses (DDRs) [109]. Besides vFLIP, two KSHV proteins expressed during the lytic cycle, vBcl2 and K7, can interfere with autophagy. vBcl2 is a homolog of cellular Bcl-2 that inhibits both apoptosis [99, 110] and autophagy [34, 35]. As its cellular counterpart, vBcl2 negatively regulates autophagy by binding Beclin 1 [34]. Always within the context of the lytic cycle, recent evidence suggests that KSHV K7 protein prevents autophagosome maturation, by interacting with Rubicon autophagic protein [111]. K7 is involved in apoptosis suppression [112, 113] while Rubicon is a subunit of the Beclin 1/UVRAG/Vps34 autophagy complex, which regulates the autophagosome maturation and the endocytic trafficking [114–116]. As shown by authors, K7 transfection in epithelial cell lines promoted a greater interaction between Rubicon and Beclin 1/UVRAG/Vps34 complex resulting in a block of Vps34 enzymatic activity and, consequently, of autophagosome maturation [111]. Interestingly, during the lytic cycle KSHV exploits autophagy to enhance its reactivation mediated by Rta immediate lytic protein [28]. Altogether, the data here reviewed suggest that KSHV has evolved several strategies to circumvent autophagy-mediated immune responses, to persist in infected hosts. Moreover, the autophagic block during latency and the autophagy induction during the lytic cycle may contribute to the pathogenesis of KSHV associated malignancies.

## 6. Concluding Remarks

A better understanding of how autophagy manipulation could influence antiviral immune response and control virus-induced tumorigenesis might help to discover strategies improving the outcome of the treatments of virus-associated malignancies. Moreover, since autophagy is activated and is involved in EBV and KSHV replication [20, 27], its manipulation could also affect the viral particle release that plays a role in *γ*-Herpesvirus-associated cancers.
